# Incremental predictive utility of a radiomics signature in a nomogram for the recurrence of atrial fibrillation

**DOI:** 10.3389/fcvm.2023.1203009

**Published:** 2023-08-11

**Authors:** Dongyan Zheng, Yueli Zhang, Dong Huang, Man Wang, Ning Guo, Shu Zhu, Juanjuan Zhang, Tao Ying

**Affiliations:** ^1^ Department of Ultrasound, Shanghai Jiao Tong University Affiliated Sixth People’s Hospital, Shanghai, China; ^2^ Department of Cardiology, Shanghai Jiao Tong University Affiliated Sixth People’s Hospital, Shanghai, China

**Keywords:** atrial fibrillation, recurrence, catheter ablation, speckle-tracking echocardiography, radiomics, nomogram

## Abstract

**Background:**

Recurrence of atrial fibrillation (AF) after catheter ablation (CA) remains a challenge today. Although it is believed that evaluating the structural and functional remodeling of the left atrium (LA) may be helpful in predicting AF recurrence, there is a lack of consensus on prediction accuracy. Ultrasound-based radiomics is currently receiving increasing attention because it might aid in the diagnosis and prognosis prediction of AF recurrence. However, research on LA ultrasound radiomics is limited.

**Objective:**

We aim to investigate the incremental predictive utility of LA radiomics and construct a radiomics nomogram to preoperatively predict AF recurrence following CA.

**Methods:**

A training cohort of 232 AF patients was designed for nomogram construction, while a validation cohort (*n* = 100) served as the model performance test. AF recurrence during a follow-up period of 3–12 months was defined as the endpoint. The radiomics features related to AF recurrence were extracted and selected to create the radiomics score (rad score). These rad scores, along with other morphological and functional indicators for AF recurrence, were included in the multivariate Cox analysis to establish a nomogram for the prediction of the likelihood of AF recurrence within 1 year following CA.

**Results:**

In the training and validation cohorts, AF recurrence rates accounted for 32.3% (75/232) and 25.0% (25/100), respectively. We extracted seven types of radiomics features associated with AF recurrence from apical four-chamber view echocardiography images and established a rad score for each patient. The radiomics nomogram was built with the rad score, AF type, left atrial appendage emptying flow velocity, and peak atrial longitudinal strain. It outperformed the nomogram building without the rad score in terms of the predictive efficacy of CA outcome and showed favorable performance in both cohorts.

**Conclusion:**

We revealed the incremental utility of a radiomics signature in the prediction of AF recurrence and preliminarily developed and validated a radiomics nomogram for identifying patients who were at high risk of post-CA recurrence, which contributed to an appropriate management strategy for AF.

## Introduction

Atrial fibrillation (AF) affects 1%–2% of the global population, and its incidence rises rapidly with advancing age (1, 2). According to longitudinal data from the Framingham Heart Study, the lifetime risk of developing AF after 55 years of age has now reached 37% (3). Catheter ablation (CA), which eliminates the initial foci of AF, has been demonstrated to be one of the most effective treatments for AF and is recommended by the European Society of Cardiology as the first-line rhythm control therapy (4, 5). Despite recent advances in CA, approximately one-third of AF patients experience recurrence after ablation (6–8). Previous studies on AF have indicated that it may lead to structural and functional remodeling of the left atrium (LA) (9). Consequently, there is interest in predicting the likelihood of recurrence by an accurate evaluation of the abnormalities of LA structure and function, which may aid in patient selection and post-CA planning (10–12).

Several LA morphological and functional changes found to be associated with AF recurrence after CA have been developed and tested, including LA volume (LAV) (13), LA diameter (LAD) (14, 15), and peak atrial longitudinal strain (PALS) by speckle-tracking echocardiography (STE) (16–18). However, the discrimination of these markers, especially morphological markers such as LAV and LAD, varies widely, and there is a lack of consensus on their strength in measuring the risk of AF recurrence (19). Therefore, the reliable identification of AF patients who will recur after CA remains a challenge. Ultrasound-based radiomics has received increasing attention in the analysis of medical imaging (20–22). It enables the description of objective but visually unrecognizable tissue heterogeneity by extracting features from high-throughput medical images to improve the accuracy of disease diagnosis and prognosis prediction (23, 24). In the field of cardiology, ultrasound radiomics has shown promise in a number of areas, including assessing cardiac remodeling and dysfunction (25) and differentiating hypertrophic and uremic cardiomyopathy from hypertensive heart disease (26), and more applications are under active research. Hence, we hypothesize that LA ultrasound radiomics analysis might have potential in the prediction of AF recurrence after CA by separating AF features. However, few prediction models integrated with LA ultrasound radiomics have been proposed to date.

To assess this hypothesis, we aim to investigate the incremental predictive utility of LA radiomics and construct and validate a nomogram by using the radiomics score (rad score) associated with AF recurrence, as well as other morphological and functional features, for preoperatively predicting recurrence following CA in AF patients.

## Materials and methods

This retrospective study was approved by the Institutional Review Board of Shanghai Sixth People's Hospital Affiliated with Shanghai Jiao Tong University School of Medicine [2021-KY-060 (K)] for a review of medical records. It was performed in accordance with the Declaration of Helsinki, and all participants provided informed consent.

### Patient selection

To ensure robust statistical power, we first estimated the required sample size. According to guidelines on the transparent reporting of a multivariable prediction model for individual prognosis or diagnosis (27), a minimum of 10 events per variable is required to develop a multivariable logistic regression–based nomogram. Thus, for a model comprising 4–5 predictors, we needed a minimum of 40–50 events. Given that previous studies have suggested that AF recurrence is expected in approximately 30% of patients following CA (6, 7), we calculated a need for at least 130–170 patients in our training cohort. Guided by these calculations, we retrieved data from 382 non-valvular AF patients, who were referred for pulmonary vein (PV) isolation with radiofrequency catheter ablation (RFA) between June 2018 and December 2022. All patients met the criteria of the 2014 guidelines for AF management, with paroxysmal AF (PAF) being self-limited within 7 days of onset and persistent AF (PeAF) lasting longer than 7 days or requiring cardioversion (28).

The patients were eligible for inclusion if they met the following criteria: (1) underwent a thorough clinical examination, including electrocardiogram (ECG), transthoracic echocardiography (TTE), transesophageal echocardiography (TEE), and contrasted-enhanced CT angiography, and (2) no previous CA treatment. The exclusion criteria for this study were the following: (1) moderate or greater structural heart disease (cardiomyopathy, congenital heart disease, and heart valve disease) (*n* = 15), (2) history of myocardial infarction (*n* = 4), (3) LA volume of >150 ml (*n* = 2), (4) left ventricular ejection fraction (LVEF) of <50% (*n* = 4), (5) history of cardiac surgery (*n* = 3), (6) uninterpretable image quality (*n* = 7), and (7) patients lost to follow-up (*n* = 9).

**Figure 1:**
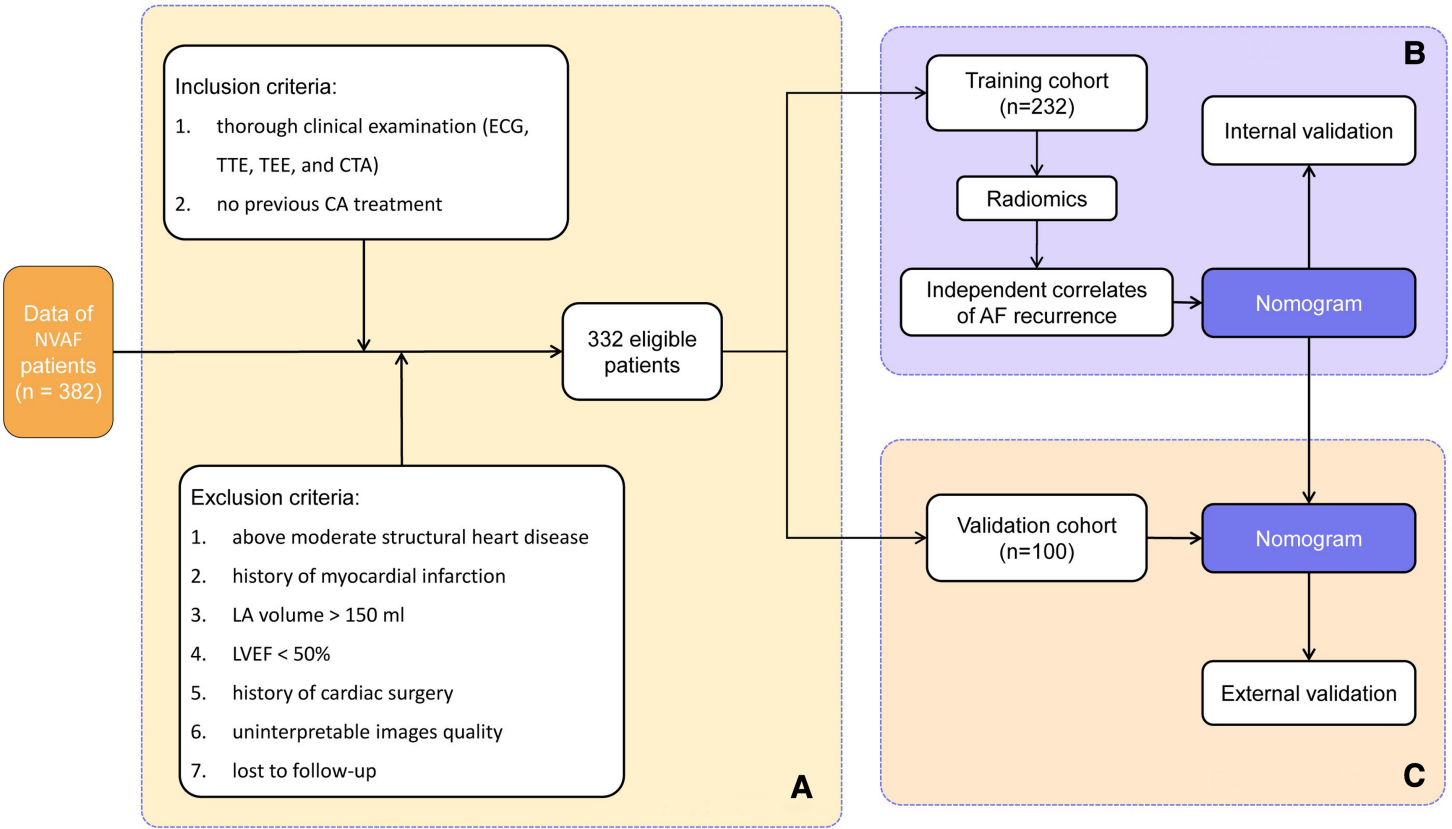
Workflow showing (A) patient selection and (B) model development and (C) model validation. NVAF, non-valvular atrial fibrillation; ECG, electrocardiogram; TTE, transthoracic echocardiography; TEE, transesophageal echocardiography; CTA, CT angiography; CA, catheter ablation; LA, left atrium; LVEF, left ventricular ejection fraction.

A total of 332 AF patients met these criteria and were enrolled in this study (226 and 106 for PAF and PeAF, respectively). The cohort was then randomly divided into 232 patients for the training cohort and 100 patients for the validation cohort in a ratio of 7:3. Figure 1 shows a detailed flowchart of this study. By querying the medical record system, we obtained the demographics and clinical characteristics of each patient, such as age, gender, body mass index (BMI), AF duration, AF type (PAF or PeAF), smoking history, hypertension, type 2 diabetes (T2DM), dyslipidemia, and current medical treatment.

### RFA procedure

Before performing the RFA procedure, a new oral anticoagulant (Rivaroxaban, Dabigatran, or Edoxaban) was used with recommended dosage uninterruptedly. Vascular access was established through the femoral veins. After the administration of intravenous heparin (activated clotting time goal of >300 s), the flexible decapolar catheter and temporary pacing electrode were placed in the coronary sinus and right ventricle apex through the left femoral vein, and then, the LA was accessed by double trans-septal punctures under x-ray fluoroscopy guiding. PV isolation was performed using radiofrequency applications, guided by a three-dimensional electroanatomic mapping system (CARTO 3, Biosense Webster, Diamond Bar, CA, USA). RFA was performed using a 3.5 mm tip irrigated ablation contact force monitoring catheter (Smarttouch, Biosense Webster, Diamond Bar, CA, USA). The procedural endpoint was defined as the elimination of electrograms at the PV antra on a circumferential mapping catheter (PentaRay, Biosense Webster, Diamond Bar, CA, USA) and the demonstration of an entrance block into the PVs. In selected patients with persistent atrial fibrillation, after the PV isolation, additional interventions such as the treatment of complex fractional atrial electrograms (CFAE) and linear ablation at the LA roof, mitral isthmus, or cavo-tricuspid isthmus were performed. The confirmation of no potential recovery for at least 30 min was validated using a PentaRay electrode (Biosense Webster, Diamond Bar, CA, USA).

All patients continued their anticoagulant treatment for 2 months following the ablation, with adjustments made depending on their individual stroke risk, and they underwent antiarrhythmic drug therapy for 3 months after surgery. Follow-up was performed at 3, 6, and 12 months. The patients were questioned about any arrhythmia-related symptoms at each visit, followed by a 12-lead ECG and 24 h Holter monitoring. AF recurrence, defined as any atrial tachyarrhythmia greater than 30 s identified by a 12-lead ECG or Holter after a 3-month blank period, signified the end of follow-up (29).

#### Image acquisition

The images for LA radiomics and STE analyses were obtained from TTE prior to AC. The imaging examinations were performed by two experienced sonographers using the EPIQ 7C ultrasound system (Philips Medical Systems, Bothell, WA, USA). The limb lead ECG was recorded while the patients were lying on the left side and breathing calmly. The imaging data of all patients were collected when monitoring the sinus rhythm. All measurements were taken in standard echocardiographic views and were performed in accordance with the recommendations of the American Society of Echocardiography (30). LVEF was obtained from the apical four-chamber and two-chamber views (4CV and 2CV) by Simpson's biplane method. The LA maximal volume index (LAVImax) was calculated from the apical 4CV and 2CV of the LA by the biplane method during the diastolic phase and then divided by body surface area. TEE was performed to check for the formation of a thrombus in the left atrial appendage (LAA), and the LAA emptying flow velocity (LAAFV) was assessed using a pulse-wave Doppler by positioning the sample volume at a length of 1 cm from the LAA orifice.

#### Two-dimensional STE

Three cine loops from 4CV and 2CV acquired with a frame rate of >50 frames/s were saved for offline analysis. To calculate the LA strain, we employed QLAB software (version 13.0, Philips), which automatically contoured the LA endocardial border at the end diastole in both the 4CV and the 2CV. Manual adjustments were made as necessary. PALS was determined by averaging the values obtained from three cardiac cycles. We excluded the LA appendage and pulmonary veins from the LA strain analysis.

### Radiomics feature extraction

**Figure 2:**
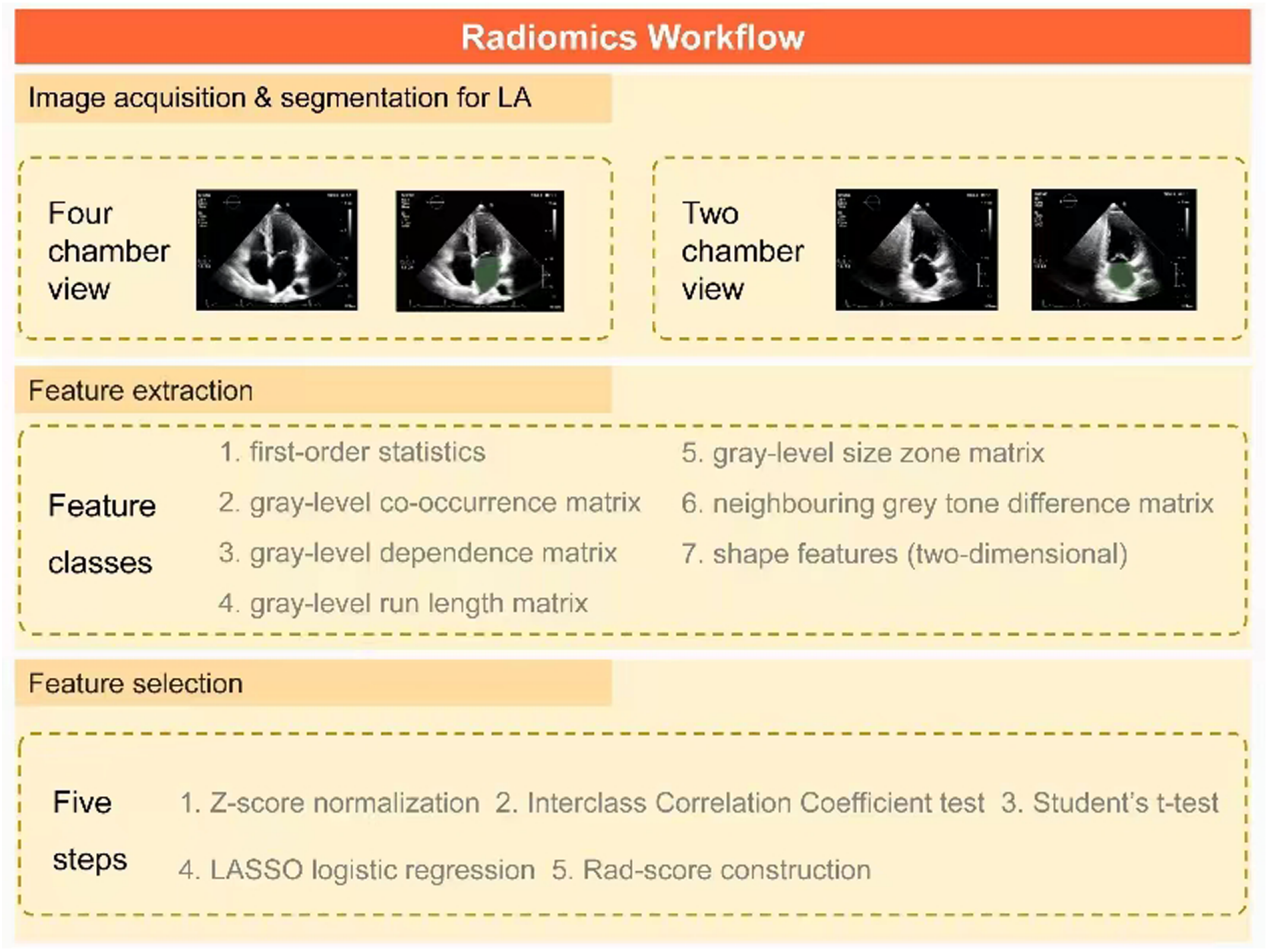
Key steps in a radiomics workflow for AF patients. First, ROIs containing LA are delineated on apical 4CV and 2CV images, respectively, to extract seven types of radiomics features. After normalization, the key radiomics features associated with AF recurrence are identified by a three-step feature selection method (interclass correlation coefficient test, Student's t-test, and LASSO logistic regression). Finally, they are integrated into the rad score using linear regression. AF, atrial fibrillation; ROI, region of interest; LA, left atrium; 4CV, four-chamber view; 2CV, two-chamber view; LASSO, least absolute shrinkage and selection operator; Rad score, radiomics score.

The process of LA radiomics analysis, as described in Figure 2, primarily involved image acquisition and segmentation as well as feature extraction and selection. This process was implemented using the 3D Slicer software (v5.0.2), which enabled a detailed LA segmentation and subsequent radiomics analysis on echocardiographic images of apical 4CV and 2CV. After the digital imaging and communications in medicine files were imported offline, two experienced sonographers manually delineated the region of interest (ROI) encompassing the LA. Utilizing the radiomics extension of the 3D Slicer, seven distinct classes of radiomics features were extracted including shape, first-order statistics, gray-level co-occurrence matrix, gray-level dependence matrix, gray-level run length matrix, gray-level size zone matrix, and neighboring gray tone difference matrix. In addition, as a crucial component of the extraction process, wavelet transform was applied, encompassing all possible combinations of high-pass (H) or low-pass (L) filters to each ROI, resulting in the following eight unique wavelet decompositions: LLH, LHL, LHH, HLL, HLH, HHL, HHH, and LLL (31).

### Rad-score development

Through *Z*-score normalization, all features extracted from the LA segmentations were rescaled. The reproducibility of the extracted features was evaluated based on interoperator tests. The interclass correlation coefficients (ICCs) of <0.8 were disregarded for further investigation because of their low repeatability. Then, each feature was compared between patients with and without AF recurrence using the Student's *t*-test. The least absolute shrinkage and selection operator (LASSO) logistic regression with a 10-fold cross validation was utilized to select the related features of AF recurrence with non-zero coefficients from those having false discovery rate (FDR)-adjusted *P*-values of <0.05 in the *t*-test. Finally, the LASSO-weighted linear combination of the selected features was utilized to develop the rad score.

### Nomogram development

In the development of the nomogram, independent correlates of AF recurrence were identified. The initial selection of predictors was carried out through univariate Cox analyses, including rad scores and other factors of AF recurrence. These selected predictors were subsequently incorporated into a multivariate Cox analysis. A radiomics nomogram was then constructed, incorporating all identified predictors along with their associated regression coefficients. To evaluate the performance of this nomogram, a two-step validation process was adopted. Initially, a comprehensive assessment was conducted using the training cohort. This was followed by an external validation, which involved applying the nomogram to the validation cohort with identical parameter estimates, thereby ensuring robust reproducibility and predictive accuracy of the model.

### Statistical analysis

The comparisons of continuous variables between the training and the validation cohorts were run using the independent-sample *t*-test or the Mann–Whitney *U* test according to the distribution of the variables (normal distribution, non-normal distribution, etc.), which was checked using the Shapiro–Wilk test. Categorical variables were compared using the chi-square test or Fisher’s exact test if the expected count in any cell was less than 5. Multivariate Cox regression analysis was used to obtain the hazard ratio (HR) and 95% confidence interval (CI) of each independent predictor that was utilized to develop the nomogram. Prior to using the Cox regression model, the validity of the proportional hazards assumption and the linearity assumption for each continuous variable against survival time were assessed using Schoenfeld residuals and Martingale residuals, respectively.

In the assessment of model performance, the receiver operating characteristic (ROC) curve was employed and the area under the curve (AUC) was utilized to evaluate the model discrimination. Comparisons between AUCs in the nomograms with and without the rad score were performed using Delong's test to assess the incremental predictive power of radiomics. The goodness of fit was evaluated by the calibration curve along with the Hosmer–Lemeshow (HL) test. Decision curve analysis (DCA) was utilized to evaluate the clinical net benefits of various threshold probabilities, thereby determining the clinical applicability of the model. Statistical processing of data was performed by using IBM SPSS Statistics (v 22.0, SPSS Inc.), MedCalc (v 19.2.1), R package (v 4.2.1), and Python (v 3.7.1).

## Results

### Patient summary

The baseline clinical and echocardiographic data of the eligible AF patients are summarized in Table 1. In the training and validation cohorts, AF recurrence rates accounted for 32.3% (75/232) and 25.0% (25/100), respectively. Only the age and BMI satisfied the normal distribution and variance homogeneity and hence compared with the *t*-test (age: *P* = 0.197 and 0.775, Shapiro–Wilk test; *P* = 0.691, Levene's test; BMI: *P* = 0.814 and 0.770, Shapiro–Wilk test; *P* = 0.559, Levene's test). Chi-square tests were applied for all categorical variables because no expected count in any cell was less than 5. There were no statistically significant differences with respect to demographics, clinical characteristics, current medical treatment, and echocardiographic parameters between the two cohorts (all FDR-adjusted *P*-values of >0.05).

**Table 1:** Comparisons of the baseline clinical and echocardiographic data between the training and the validation cohorts.

Variable		Training cohort (*n* = 232)	Validation cohort (*n* = 100)	*P*-value	FDR-adjusted *P*-value
Age, year	61.56 ± 6.33	61.01 ± 6.49	0.475	0.687^a^
Gender, *n* (%)	Male	147 (63.4%)	67 (67.0%)	0.525	0.687^b^
	Female	85 (36.6%)	33 (33.0%)		
BMI, kg/m^2^		23.55 ± 3.13	23.11 ± 3.36	0.250	0.605^a^
Hypertension, *n* (%)		107 (46.1%)	53 (53.0%)	0.250	0.605^b^
T2DM, *n* (%)		35 (15.1%)	18 (18.0%)	0.506	0.687^b^
Dyslipidemia, *n* (%)		66 (28.4%)	26 (26.0%)	0.647	0.786^b^
Smoking, *n* (%)		60 (25.9%)	32 (32.0%)	0.252	0.605^b^
AF type	PAF	154 (66.4%)	72 (72.0%)	0.314	0.605^b^
	PeAF	78 (33.6%)	28 (28.0%)		
AF duration, year		3 (2–4)	3 (2–4)	0.943	0.943^c^
Antiarrhythmic drugs failure, *n* (%)		156 (67.2%)	62 (62.0%)	0.356	0.605^b^
AF recurrence, *n* (%)		75 (32.3%)	25 (25.0%)	0.182	0.605^b^
Time to recurrence	<6 months	19 (25.3%)	9 (36.0%)	0.304	0.605^b^
	≥6 months	56 (74.7%)	16 (64.0%)		
LVEF, %		57.4 (54.2–60.6)	57.3 (54.1–60.7)	0.916	0.943^c^
LAD, mm		42.0 (38.4–45.9)	43.6 (39.0–46.2)	0.323	0.605^c^
LAVImax, ml/m^2^		38.2 (32.8–43.4)	36.7 (32.7–42.4)	0.288	0.605^c^
LAAFV, cm/s		46.8 (37.2–57.4)	47.5 (39.9–52.8)	0.886	0.943^c^
PALS, %		24.6 (21.5–28.3)	26.3 (22.3–31.1)	0.142	0.605^c^

Variables are expressed as median (interquartile range) if they are not normally distributed.

FDR, false discovery rate; BMI, body mass index; T2DM, type 2 diabetes; AF, atrial fibrillation; PAF, paroxysmal atrial fibrillation; PeAF, persistent atrial fibrillation; LVEF, left ventricular ejection fraction; LAD, left atrial diameter; LAVImax, left atrium maximal volume index; LAAFV, left atrial appendage emptying flow velocity; PALS, peak atrial longitudinal strain.

^a^ For an independent-sample *t*-test.

^b^ For a chi-square test.

^c^ For a Mann–Whitney *U* test.

### Radiomics analyses

**Figure 3:**
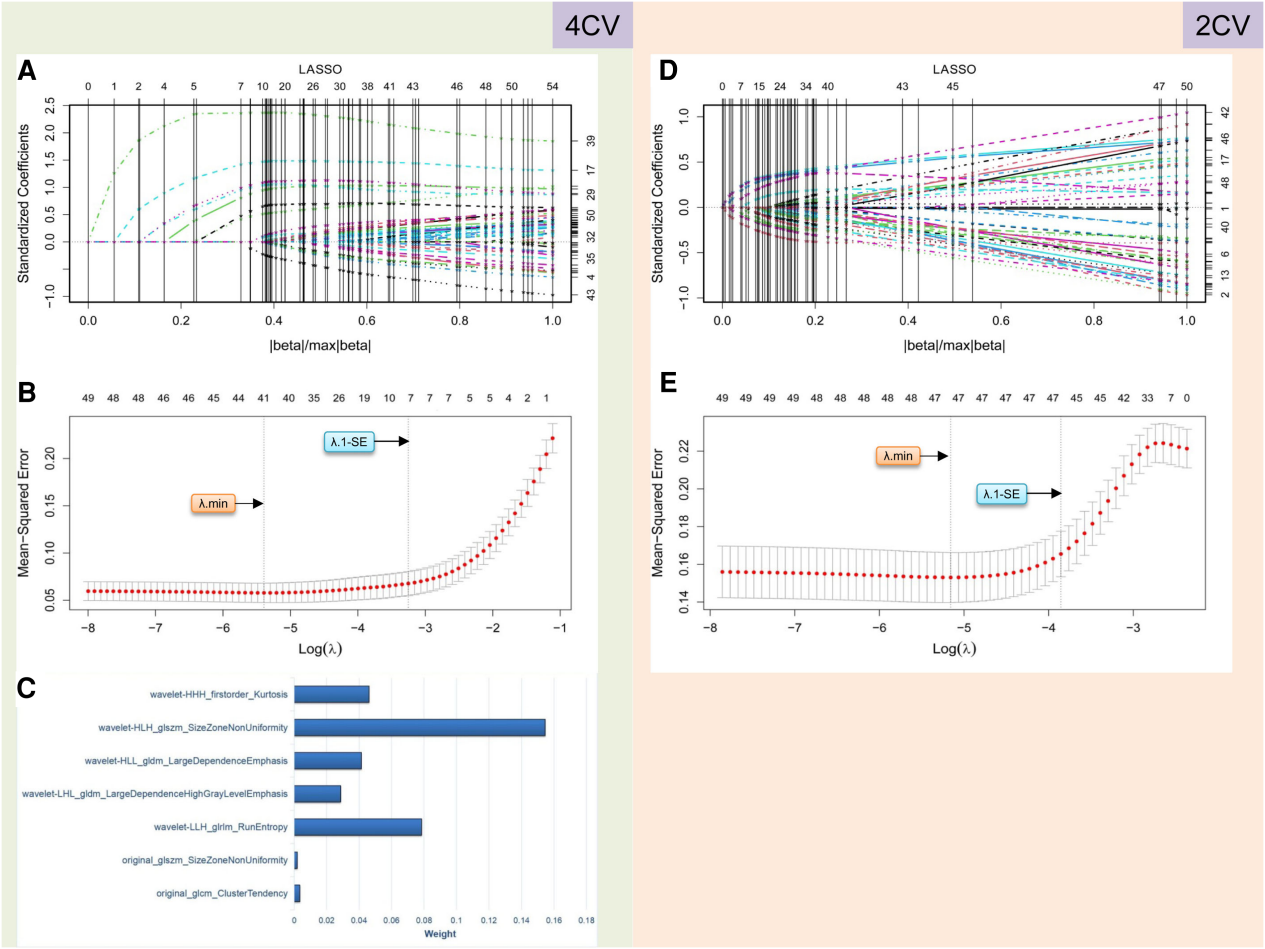
Radiomics feature selection performed using LASSO logistic regression for establishing the rad score. (**A**–**C**) and (**D**–**E**) describe the results of radiomics analysis for 4CV and 2CV images, respectively. In 4CV analysis, seven features are selected by identifying the best *λ* with *λ*.1-SE in the LASSO model (**A**,**B**), and their weights are listed in (**C**). In 2CV analysis, no features can be selected with the optimal *λ* values (**D**,**E**). LASSO, least absolute shrinkage and selection operator; 4CV, four-chamber view; 2CV, two-chamber view; *λ*, penalty regularization parameter; *λ*.min, minimum criteria; *λ*.1-SE, 1-standard error of the minimum criteria.

A total of 837 radiomics features were extracted from the 4CV and 2CV images of each patient, respectively. We performed feature selection in the training cohort of 238 patients to establish the rad score. In 4CV images, we selected 632 features (75.5%) with an intraobserver ICC of ≥0.8 among the normalized features. Fifty-four features associated with AF recurrence were initially screened using Student's *t*-test. Finally, seven features associated with AF recurrence with non-zero coefficients were selected using the LASSO regression model (Figures 3A,B). The rad score was developed from these features and their corresponding coefficients (Figure 3C). Its calculation is presented in Supplementary Appendix 1. In 2CV images, 663 features (79.2%) with an intraobserver ICC of ≥0.8 were arranged in the subsequent *t*-test comparisons, which yielded 50 features associated with the recurrence. However, according to the LASSO regression screening result, there were no features with non-zero coefficients linked to AF recurrence (Figures 3D,E), indicating that the radiomics of the 2CV images was ineffective at predicting AF recurrence. Ultimately, the median rad score in the training and validation cohorts was 0.22 (interquartile range: 0.15–0.48) and 0.20 (interquartile range: 0.14–0.25), respectively. This difference was not statistically significant (FDR-adjusted *P*-values = 0.593).

### Radiomics nomogram development

To develop the radiomics nomogram, the clinical characteristics and echocardiographic parameters of the patients in the training cohort were assessed using univariate Cox regression followed by multivariate Cox regression. All variables satisfied the proportional hazards assumption (Supplementary Figure S1). Among them, except for BMI, LAD, and LVEF, the other continuous variables met the linear assumption (Supplementary Figure S2). Table 2 illustrates the Cox regression analyses of the predictors associated with AF recurrence. It reveals that PeAF, lower LAAFV, and lower PALS are independent correlates of AF recurrence.

**Table 2:** Cox regression analyses for the predictors associated with AF recurrence post-CA.

Variable		Univariate Cox regression			Multivariate Cox regression		
		*P*	HR	95% CI	*P*	HR	95% CI
Age		0.104	1.030	0.994–1.067			
Gender	Female	Reference					
	Male	0.489	1.185	0.733–1.914			
Hypertension		0.386	1.222	0.777–1.921			
T2DM		0.447	1.261	0.693–2.293			
Dyslipidemia		0.672	0.895	0.537–1.494			
Smoking		0.174	1.398	0.861–2.271			
AF type	PAF	Reference			Reference		
	PeAF	0.007	1.878	1.192–2.957	0.033	1.647	1.041–2.606
AF duration		0.610	0.960	0.820–1.124			
Antiarrhythmic drugs failure		0.482	0.845	0.527–1.353			
LAVImax		0.002	1.049	1.018–1.081	0.128	1.042	0.972–1.067
LAAFV		0.007	0.979	0.964–0.994	0.003	0.978	0.963–0.992
PALS		<0.001	0.889	0.850–0.931	<0.001	0.887	0.847–0.928

HR, hazard ratio; CI, confidence interval; T2DM, type 2 diabetes; AF, atrial fibrillation; PAF, paroxysmal atrial fibrillation; PeAF, persistent atrial fibrillation; LAVImax, left atrium maximal volume index; LAAFV, left atrial appendage emptying flow velocity; PALS, peak atrial longitudinal strain.

**Figure 4:**
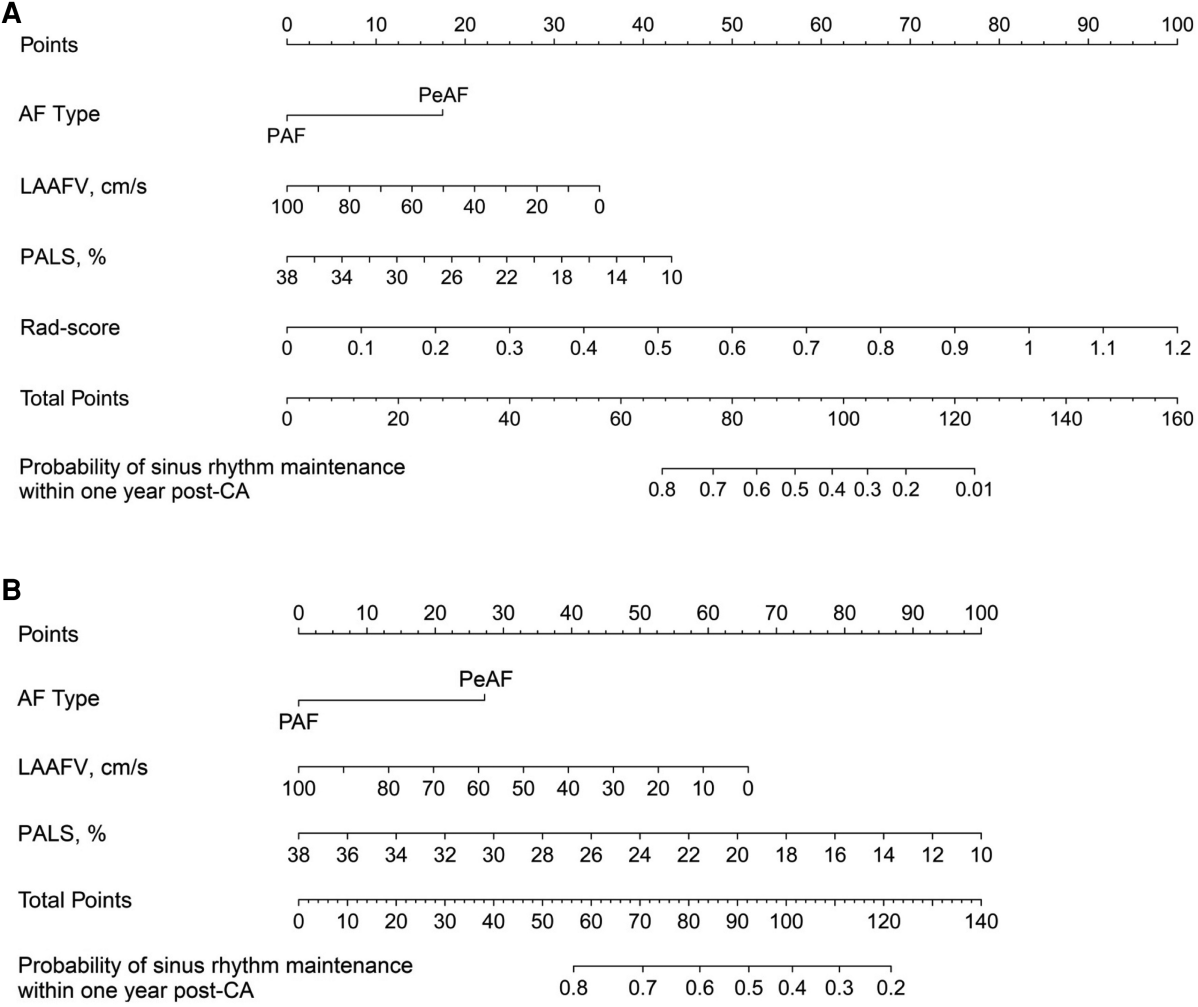
Nomograms developed for predicting the likelihood of sinus rhythm maintenance within 1 year following CA. (**A**) illustrates a radiomics nomogram, where each selected predictor, inclusive of the rad score, is assigned a score on the points scale situated at the top of the nomogram. The sum of these scores is then matched with the bottom scale, estimating the probability of sinus rhythm maintenance within 1 year after CA. As a comparison, (**B**) represents a clinical nomogram. This is designed utilizing the same set of clinical predictors as those in the radiomics nomogram, excluding the rad score. In line with the methodology of the radiomics nomogram, each predictor in the clinical nomogram is allocated a score on the points scale, and the cumulative scores serve to predict the likelihood of maintaining the sinus rhythm. AF, atrial fibrillation; PAF, paroxysmal atrial fibrillation; PeAF, persistent atrial fibrillation; LAAFV, left atrial appendage emptying flow velocity; PALS, peak atrial longitudinal strain; CA, catheter ablation.

Using the chosen predictors and the rad score, we developed a radiomics nomogram, which enabled a preoperative estimation of the likelihood of sinus rhythm maintenance within 1 year following CA (Figure 4A). In order to provide a comparative perspective, a clinical nomogram was also constructed. This was done using the same selected predictors, but without including the rad score (Figure 4B).

### Model validation

**Figure 5:**
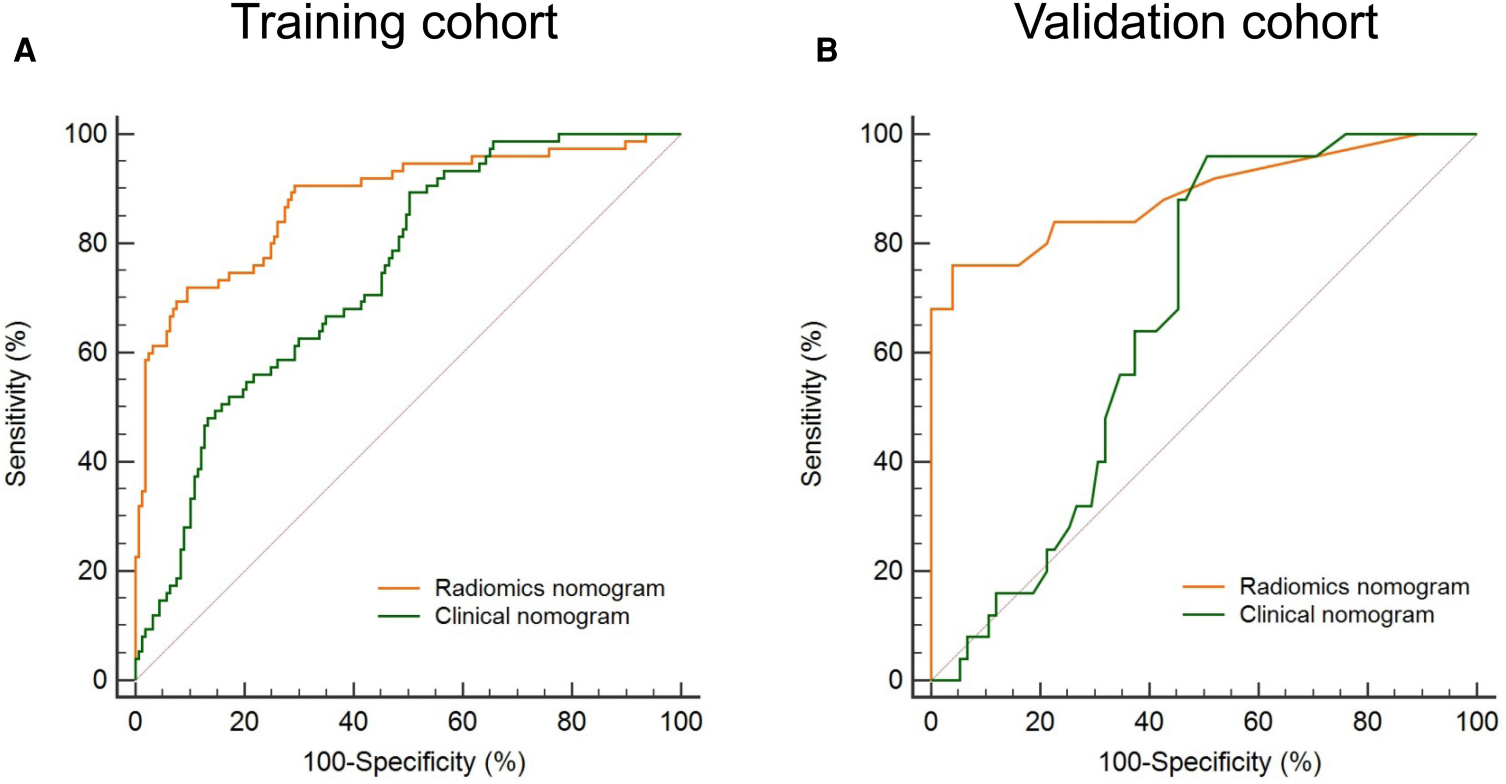
Evaluation of the model discrimination in the training and validation cohorts. The discrimination of a radiomics nomogram and a clinical nomogram (building without the rad score) is indicated by orange and green lines, respectively. The discrimination of the radiomics nomogram is higher than that of the clinical nomogram both in the training (**A**) and in the validation (**B**) cohort. ROC, receiver operating characteristic; rad score, radiomics score.

The Bootstrap method was used to resample 232 AF patients and run 1,000 times to obtain internal validation. Figure 5 compares the ROC curves of the radiomics nomogram and the clinical nomogram in the training and validation cohorts. Compared with the clinical nomogram, the discrimination rate of the radiomics nomogram significantly improved both in the training [AUC: 0.879 (95% CI: 0.830–0.918) vs. 0.744 (95% CI: 0.683–0.799); *P* < 0.001] and in the validation cohorts [AUC: 0.889 (95% CI: 0.811–0.943) vs. 0.667 (95% CI: 0.566–0.758); *P* < 0.001]. This indicated that the radiomics nomogram was more effective than the nomogram without the rad score in both cohorts, highlighting the incremental role of radiomics in prognostic prediction.

**Figure 6:**
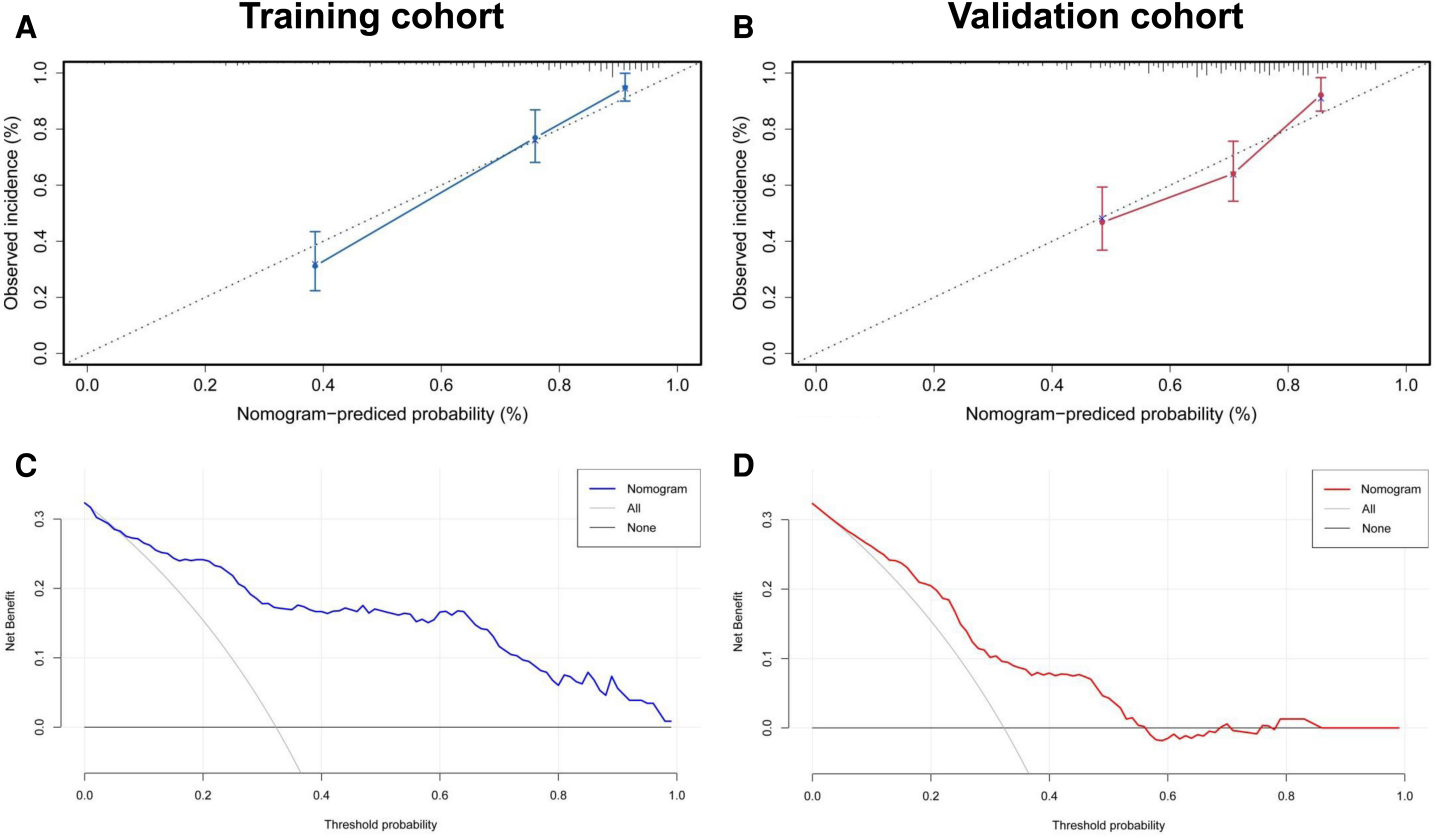
Evaluation of calibration and clinical utility of a radiomics nomogram in the training and validation cohorts. The calibration curves of the radiomics nomogram in the training (**A**) and validation (**B**) cohorts are close to the ideal line, indicating good agreement. The DCA curves for predicting the outcome of CA in the training (**C**) and validation (**D**) cohorts reveal that using the radiomics nomogram to predict the outcome within 1 year after CA adds more net benefit than the treat-all or treat-none scheme. CA, catheter ablation; DCA, decision curve analysis.

Figure 6 summarizes the calibration and clinical application of the radiomics nomogram. The calibration curves showed good agreement between the nomogram-predicted and the actual risk of post-CA recurrence in the training and validation cohorts. The HL test yielded non-significant *P*-values of 0.652 and 0.426 in both cohorts, suggesting a good fit for the nomogram. The DCA plots showed that using the radiomics nomogram to predict the outcome added more net benefit than the treat-all or treat-none scheme, suggesting the clinical benefit of the nomogram.

## Discussion

Since RFA is not a harmless procedure, an accurate estimation of the probability of sinus rhythm maintenance within 1 year after surgery aids in improving the outcomes of the ablation. In the present study, we preliminarily developed and validated a nomogram by integrating the rad score with regular prognostic markers (AF type, LAAFV, and PALS) to preoperatively determine the likelihood of experiencing AF recurrence. In both the training and the validation cohorts, this nomogram outperformed the nomogram built without the rad score and showed satisfied discrimination, calibration, and clinical utility, implying the potential in AF management.

As techniques for non-invasive assessment of LA structure and function advance, they become good predictors of AF recurrence after ablation (32, 33). The mechanisms underlying these studies are similar: LA structural and functional remodeling may lead to increased pressure and volume overload, resulting in changes in myocyte hypertrophy and interstitial fibrosis (9, 34). A more fibrotic LA triggers areas of slow conduction and altered repolarization kinetics, shifting the focus of AF initiation and maintenance from the pulmonary veins to the LA and requiring additional substrate ablation (35). Therefore, RFA may be insufficient in such patients, leading to an increased rate of recurrence.

PALS is designed to assess the LA reservoir, conduit, and booster pump functions (36, 37). It is superior to conventional echocardiographic measurements and could be a reliable predictor of AF recurrence, as it reflects LA myocardial compliance and fibrosis (38, 39). Lower PALS may imply subclinical dysfunction of the atrial myocardium, which may be associated with a higher likelihood of post-CA recurrence. Nevertheless, it is difficult to assess atrial subclinical changes in the LA structure using both LAD and LAV in the absence of LA enlargement. This leads to a significant variation in the accuracies of prediction models for AF recurrence based on LA structure and function (19). However, it is anticipated that radiomics could overcome this shortcoming.

Radiomics has grown in popularity over the past 10 years because of its ability to assess heterogeneity using mathematical algorithms for clinical diagnosis and prognostic assessment (40). It provides a non-invasive approach to make the microcosmic details that are assessed inaccessible to visual interpretation (23), meaning it is a promising tool for predicting AF recurrence. In this study, we employed radiomics signatures for the first time to predict the likelihood of AF recurrence within 1 year after ablation. After screening by LASSO regression, the seven features most associated with AF recurrence were ultimately selected in apical 4CV images. However, although radiomics-based image analysis is as effective as or even better than human interpretation, it remains challenging to interpret the pathophysiological significance of these features (41, 42). The solution is to develop a multiparameter model to estimate the results. Therefore, we combined the rad score with a nomogram to improve its accuracy in predicting AF recurrence.

Our nomogram offered an intuitive approach to determine who was at high risk of AF recurrence. In addition to the rad score and PALS, the components of the nomogram also included AF type and LAAFV. Consistent with prior studies, PeAF was found to be an independent predictor of AF recurrence (43, 44). This may be attributed to the higher degree of atrial remodeling in PeAF patients. LAAFV is regarded as a reliable predictor of AF recurrence because it reflects the comprehensive LAA hemodynamics and the severity of LA remodeling (45). Similar to the study by Kim et al. (46), we found that patients with decreased LAAFV before ablation were more likely to experience AF recurrence. Therefore, it is reasonable to include these regular predictors in the nomogram.

Including our model, recent advances in radiomics-inclusive models have exhibited increased accuracy in predicting post-ablation AF recurrence (44, 47). These models seem to surpass traditional clinical prediction models (48–51). Even models utilizing advanced machine learning techniques are not immune to the limitations of relying purely on clinical indicators (48, 49). The predictive performance of our model aligns closely with that of Yang et al. (47), and our model outperforms that of Labarbera et al. (44). The limited accuracy of the latter model may result from an emphasis on conventional clinical factors, without the incorporation of quantifiable imaging parameters. Notably, in this study, we employed echocardiography data for our model instead of cardiac CT, which increases its potential for broader clinical applications. Within our model, a patient would have a greater than 50% chance of experiencing AF recurrence if their total score was more than 90 points. For instance, a 60-year-old PeAF male with 50 cm/s LAAFV, 26% PALS, and 0.6 rad score would have a total score of roughly 106 points on the nomogram, denoting a 30% chance of maintaining sinus rhythm within 1 year following CA. This necessitates more informed decision-making. The radiomics nomogram demonstrated a good capacity for predicting the likelihood of AF recurrence, with satisfied discrimination, calibration, and clinical utility. It outperformed the nomogram with the same selected predictors, but without the inclusion of the rad score. This suggests that radiomics, by providing objective and quantifiable imaging parameters, can enhance the predictive accuracy of AF recurrence, emphasizing the value of radiomics in the domain of clinical prediction.

Nevertheless, there are several limitations in our study that should be acknowledged. Primarily, our study was conducted in a single center with a limited sample size, using a specific ultrasound scanner. This could introduce interobserver variability due to differences arising from equipment or operators, potentially impacting the effectiveness of radiomics analysis. In addition, our study may have overlooked asymptomatic AF episodes and it did not include some potential AF recurrence risk factors such as N-terminal pro-brain natriuretic peptide and obstructive sleep apnea (52, 53). It is also worth mentioning that the reproducibility and validation of diverse radiomics techniques have yet to be standardized, which means alterations at any stage might affect the ultimate features and outcomes. Despite the promising performance of our model, caution is advised in interpreting the predictions, considering the relatively early stage of the clinical application of radiomics. Future research should aim at overcoming these limitations by conducting larger, prospective cohort studies across multiple medical centers. The implementation of inter-rater reliability tests for ensuring consistency among different ultrasound readings should also be considered, because this could enhance the generalizability and accuracy of the radiomics nomogram, thus improving its clinical utility in predicting AF recurrence.

## Conclusion

This study revealed that LA radiomics signatures shared the potential to improve the precision of predicting AF recurrence. Furthermore, the established radiomics nomogram, which underwent several tests to demonstrate strong predictive properties, was shown to be helpful for the preoperative prediction of the likelihood of AF recurrence within 1 year after CA. It provides more accurate information for patient education and postoperative care in management strategies for AF.

## Data Availability

The original contributions presented in the study are included in the article/**Supplementary Material**, further inquiries can be directed to the corresponding authors.

## Supplementary material

Supplementary Figure S1 Schoenfeld residuals plots for assessing the validity of proportional hazard assumption. The proportional hazards assumption is met for age (A), gender (B), BMI (C), hypertension (D), T2DM (E), dyslipidaemia (F), smoking history (G), AF type (H), AF duration (I), antiarrhythmic drugs failure (J), LVEF (K), LAD (L), LAVImax (M), LAAFV (N), and PALS (O). BMI: body mass index, T2DM: type 2 diabetes, AF: atrial fibrillation, LVEF: left ventricular ejection fraction, LAD: left atrial diameter, LAVImax: left atrium maximal volume index, LAAFV: left atrial appendage emptying flow velocity, PALS: peak atrial longitudinal strain.

Supplementary Figure S2 Martingale residual plots for assessing the linearity assumption of continuous variables against survival time. Overall, there is a linear trend in age (A), AF duration (C), LAVImax (F), LAAFV (G), and PALS (H), while BMI (B), LVEF (D), and LAD (E) do not met the linear assumption. BMI: body mass index, AF: atrial fibrillation, LVEF: left ventricular ejection fraction, LAD: left atrial diameter, LAVImax: left atrium maximal volume index, LAAFV: left atrial appendage emptying flow velocity, PALS: peak atrial longitudinal strain.
