# Supplementary material for: Incremental predictive utility of a radiomics signature in a nomogram for the recurrence of atrial fibrillation
Source: Front Cardiovasc Med. 2023 Aug 11;10:1203009. doi: 10.3389/fcvm.2023.1203009 (PMC10451088; doi:10.3389/fcvm.2023.1203009)
Supplement: Supplementary file 1 [file Table_1.docx]

**Supplementary**

**Appendix 1**

Rad-score = 0.3232759+0.003488×original_glcm_ClusterTendency

+0.001989×original_glszm_SizeZoneNonUniformity

+0.078601×wavelet-LLH_glrlm_RunEntropy

+0.028629×wavelet-LHL_gldm_LargeDependenceHighGrayLevelEmphasis

+0.0415253×wavelet-HLL_gldm_LargeDependenceEmphasis

+0.1545547×wavelet-HLH_glszm_SizeZoneNonUniformity

+0.046174×wavelet-HHH_firstorder_Kurtosis

Wavelet transform applies all possible combinations of high-pass (H) or low-pass (L) filters (LLH, LHL, LHH, HLL, HLH, HHL, HHH, LLL), resulting in 8 decompositions.
