# Supplementary figures and images for: Incremental predictive utility of a radiomics signature in a nomogram for the recurrence of atrial fibrillation
Source: Front Cardiovasc Med. 2023 Aug 11;10:1203009. doi: 10.3389/fcvm.2023.1203009 (PMC10451088; doi:10.3389/fcvm.2023.1203009)

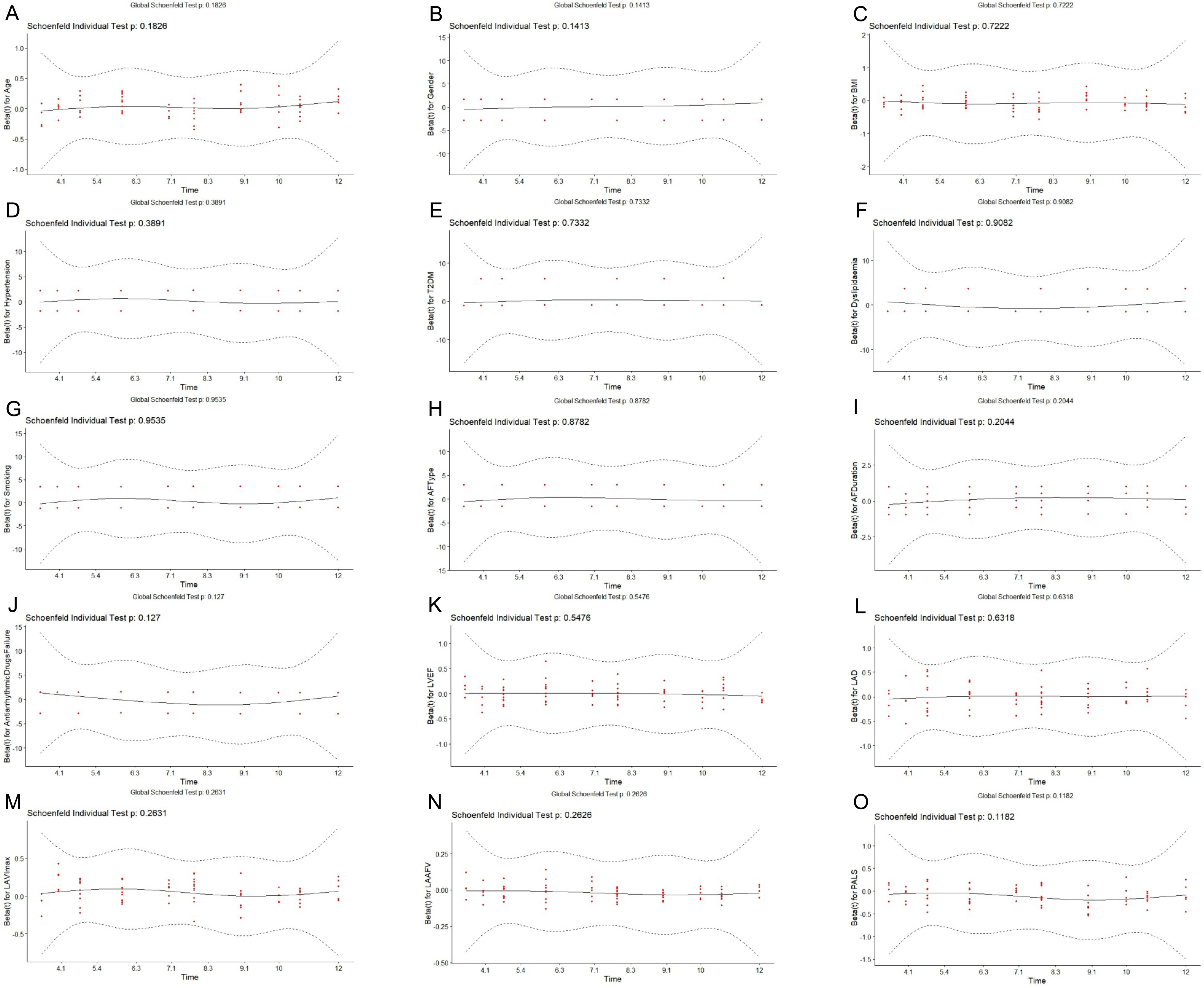

Supplement: Supplementary Figure S1 — Schoenfeld residuals plots for assessing the validity of proportional hazard assumption. The proportional hazards assumption is met for age (A), gender (B), BMI (C), hypertension (D), T2DM (E), dyslipidaemia (F), smoking history (G), AF type (H), AF duration (I), antiarrhythmic drugs failure (J), LVEF (K), LAD (L), LAVImax (M), LAAFV (N), and PALS (O). BMI: body mass index, T2DM: type 2 diabetes, AF: atrial fibrillation, LVEF: left ventricular ejection fraction, LAD: left atrial diameter, LAVImax: left atrium maximal volume index, LAAFV: left atrial appendage emptying flow velocity, PALS: peak atrial longitudinal strain. [file Image_1.jpeg]

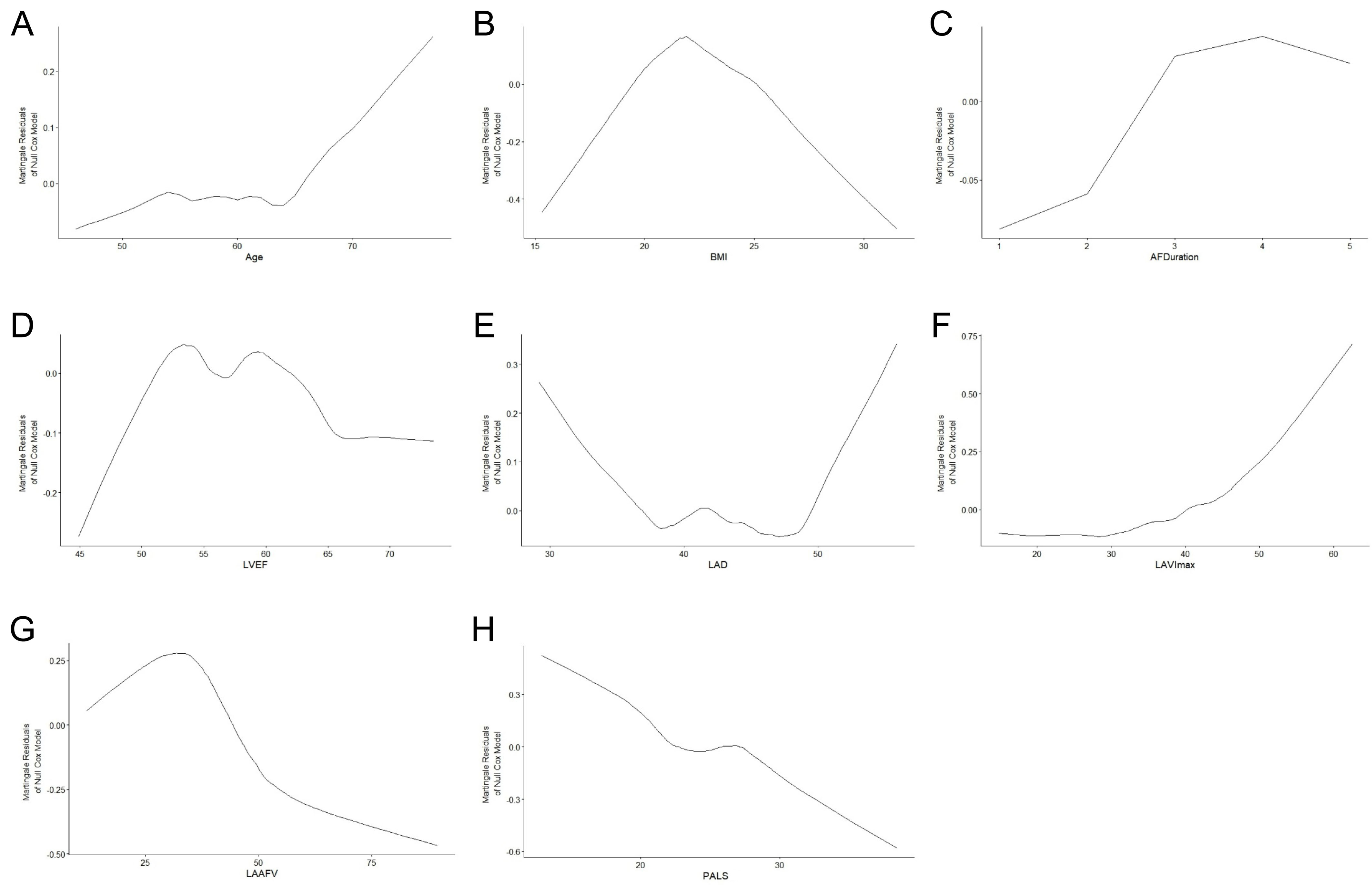

Supplement: Supplementary Figure S2 — Martingale residual plots for assessing the linearity assumption of continuous variables against survival time. Overall, there is a linear trend in age (A), AF duration (C), LAVImax (F), LAAFV (G), and PALS (H), while BMI (B), LVEF (D), and LAD (E) do not met the linear assumption. BMI: body mass index, AF: atrial fibrillation, LVEF: left ventricular ejection fraction, LAD: left atrial diameter, LAVImax: left atrium maximal volume index, LAAFV: left atrial appendage emptying flow velocity, PALS: peak atrial longitudinal strain. [file Image_2.jpeg]
